# Effectiveness of Ozonated Water on Gingivitis: A Systematic Review

**DOI:** 10.7759/cureus.61006

**Published:** 2024-05-24

**Authors:** Priyanka Bansode, Satyalakshmi K, Sathyanath D, Shrikanth Muralidharan

**Affiliations:** 1 Dentistry, Private Practice, Pune, IND; 2 Naturopathy and Yoga, National Institute of Naturopathy, Pune, IND; 3 Research, National Institute of Naturopathy, Pune, IND

**Keywords:** rct, clinical trials, review, ozonated water, gingival bleeding

## Abstract

Ozone has been used as an antibacterial agent for various purposes in healthcare. The use of ozone in dental practice is also well-established. Its utilization as a mouth rinse needs to be explored for further application in clinical practice, especially for cases of gingivitis, a common complaint. This systematic review aims to analyze the literature on the effects of ozonated water in managing gingival inflammation and bleeding across diverse populations. A systematic search adhering to Preferred Reporting Items for Systematic Reviews and Meta-Analyses (PRISMA) guidelines was conducted in PubMed, MEDLINE, Embase, and Cochrane. Studies published between January 2012 and December 2023 employing ozonated water as a treatment for gingivitis or gingival bleeding were included. Five studies met the inclusion criteria, demonstrating the varied efficacy of ozonated water. While some studies showed promising results in reducing bleeding and gingival inflammation, others indicated limitations compared to chlorhexidine. Methodological heterogeneity and lack of standardization were notable. The evidence suggests potential benefits of ozonated water in managing gingival inflammation and bleeding, but methodological variations hinder conclusive findings. Long-term studies with larger sample sizes and standardized protocols are needed to establish the effectiveness of ozonated water as an adjunctive therapy for gingival health.

## Introduction and background

Oral diseases represent a significant global health concern, with a well-established bidirectional relationship between systemic health and oral cavity status [1]. Conditions like diabetes mellitus, cardiovascular diseases, and respiratory disorders can manifest initial symptoms within the oral cavity, highlighting the critical link between oral and overall health. Therefore, maintaining optimal oral health is crucial for preventing and promoting overall well-being [1]. Gingivitis and periodontitis are inflammatory oral diseases triggered by the accumulation of microbial biofilms on tooth surfaces, commonly called dental plaque [2,3]. Gingivitis signifies the initial inflammatory response of oral tissues to dental plaque, characterized by gingival redness, edema, and bleeding on probing [2]. When left untreated, gingivitis can progress to periodontitis, a more severe condition involving progressive alveolar bone loss and potential tooth loss [4]. Historically, dental interventions focused primarily on invasive treatments rather than preventive measures. However, contemporary approaches emphasize the importance of preventive strategies for optimal oral health [1]. Plaque management, a cornerstone of preventive oral care, relies on mechanical procedures and specific chemical agents.

 While tooth brushing is a widely recommended mechanical method, chemical approaches often use anti-plaque agents containing chlorhexidine or alcohol [5]. However, the emergence and increasing prevalence of antimicrobial resistance against these conventional agents necessitate exploring alternative strategies [5]. Ozone, a naturally occurring trioxygen gas known for its protective role against ultraviolet radiation, also possesses noteworthy medicinal properties [6]. Notably, ozone dissolved in water decomposes rapidly into highly reactive hydroxyl radicals with potent oxidizing capabilities [7]. These properties confer ozone with diverse therapeutic effects, including bactericidal, virucidal, fungicidal, immune-modulatory, and anti-inflammatory benefits, making it an attractive option for various dental applications [6]. Gingivitis prevalence increases in individuals with predisposing factors, such as diabetes and smoking [8]. With its broad-spectrum antimicrobial activity and lower toxicity than conventional agents, ozonated water presents a promising alternative for managing gingivitis [8]. This systematic review aims to comprehensively evaluate the existing literature on the efficacy of ozonated water, specifically focusing on its potential to manage gingival inflammation and bleeding in diverse populations.

## Review

Materials and methods

**Figure 1:**
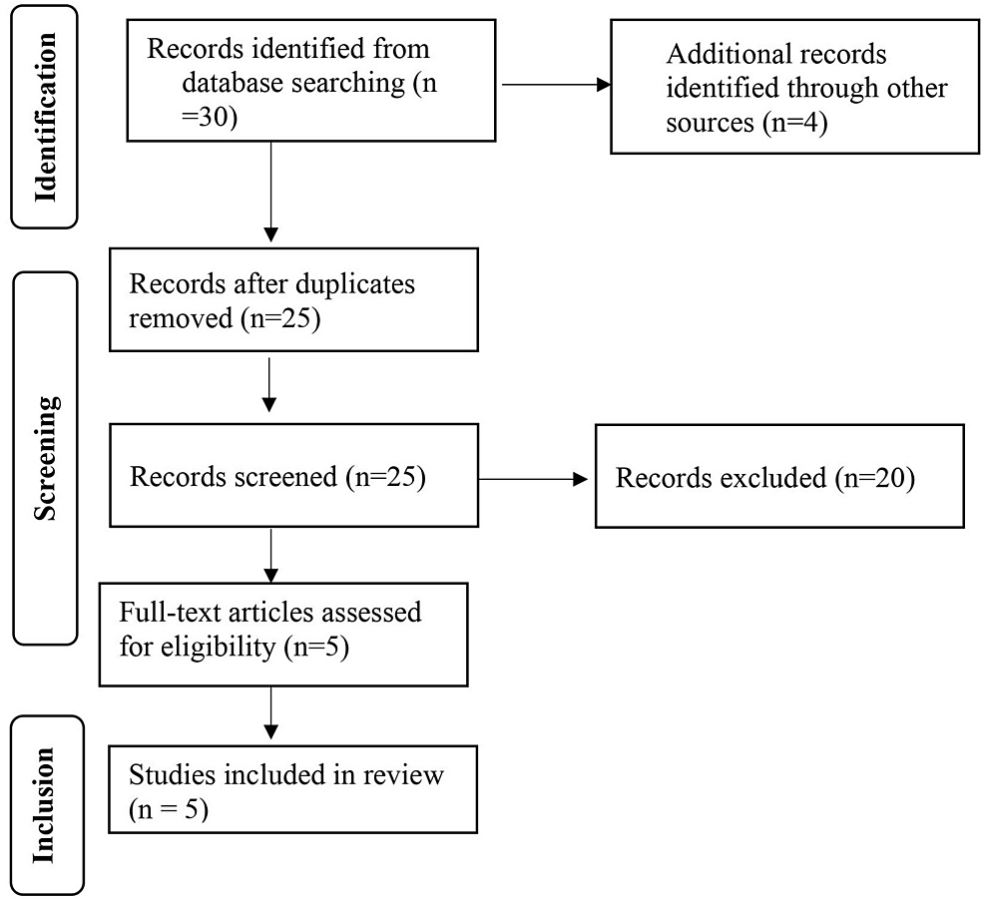
Preferred Reporting Items for Systematic Reviews and Meta-Analyses (PRISMA) flow chart

We conducted a thorough search following Preferred Reporting Items for Systematic Reviews and Meta-Analyses (PRISMA) guidelines. We scoured major databases like PubMed, MEDLINE, Embase, and Cochrane from their start dates to December 2023. To find the most relevant studies, we used a search strategy combining medical terms and keywords for "ozonated water," "gingivitis," and "gingival bleeding." We only included studies published between 2012 and 2023. Figure 1 shows our selection process (PRISMA flow diagram).

Studies included human participants diagnosed with gingivitis or gingival bleeding. The intervention of interest was ozonated water treatment. We compared its effectiveness against chlorhexidine mouthwash, placebo, or no treatment (control). Studies assessed outcomes like gingival index, plaque index, bleeding on probing (BOP), or full-mouth bleeding score (FMBS). Only randomized controlled trials (RCTs) or clinical trials were considered. We excluded studies not published in English, animal studies, reviews, editorials, and case reports. Full texts of studies were retrieved for further evaluation. Reference lists were also checked for additional relevant articles. All identified articles underwent final review by all authors, resulting in the selection of five that met our criteria. Data extraction was performed by the primary author, who meticulously reviewed the full text of each selected article. Microsoft Excel (Microsoft Corporation, USA) facilitated data organization and analysis. Due to potential variations in ozone concentration, delivery methods, and outcome measures, a meta-analysis was not feasible.

Results

The present systematic review amalgamates findings from five studies, as shown in Table 1.

**Table 1:** Data extraction from the selected five studies

Year	Author	Time	Controls' sample size	Cases' sample size	Study type	Country of study	Index	Baseline gingival scores of cases	Baseline gingival scores of controls	Post-intervention gingival scores of cases	Post-intervention gingival scores of controls
2019	Cosola et al. [9]	1 minute	15	15	Randomized clinical trial	Italy	Full mouth bleeding index	32.8 ± 8.85	31.5 ± 15.6	1.22 ± 4.00	-6.84 ± 4.00
2022	Talasani et al. [10]	1 minute	10	10	Randomized clinical trial	India	Plaque index, gingival index, full mouth bleeding index	1.7	1.8	0.8	0.3
2017	Parkar et al. [11]	1 minute	18	18	Parallel, controlled	India	Plaque index, gingival index	1.92 ± 0.32	1.58 ± 0.29	0.66 ± 0.25	0.46 ± 0.14
2017	Jose et al. [12]	1 minute	28	28	Split mouth longitudinal	India	Plaque index, gingival index, full mouth bleeding index	1.495	1.522	1.147	1.42
2022	Tecco et al. [13]	100 seconds	12	18	Randomized clinical trial	Italy	Plaque index, full mouth bleeding index	12 ± 10.13	9.29 ± 11.38	3.0 ± 3.06	8.57 ± 10.34

All studies employed an RCT design except one split-mouth longitudinal study [12]. Treatment duration varied across studies, ranging from one minute to unspecified durations. Ozone concentration was not reported in two studies [11,12]. The primary outcomes assessed were plaque index (PI), gingival index (GI), and full-mouth bleeding score (FMBS). The studies presented mixed findings regarding the efficacy of ozone-based interventions compared to control groups (placebo, no treatment, or chlorhexidine mouthwash). One RCT from Italy reported a statistically significant reduction in FMBS following one-minute ozone treatment compared to baseline (p = 1.22) [9]. Another RCT in India found similar efficacy for ozonated water compared to chlorhexidine in reducing PI and GI [11]. However, a separate Indian RCT demonstrated that chlorhexidine was more effective than ozonated water in reducing PI and GI [10]. The split-mouth longitudinal study did not observe a significant reduction in gingival inflammation with ozone [12]. Finally, an additional RCT in Italy with pregnant women showed decreased bleeding on probing (BOP) following a 100-second ozone treatment, although the specific treatment parameters were not reported [13].

Discussion

This systematic review identified five studies investigating the efficacy of ozonated water for gingivitis and gingival bleeding. The review highlights promising findings, with some studies demonstrating significant reductions in bleeding scores following ozone treatment compared to controls [9,13]. These findings align with the known biological properties of ozone, including its potential to improve tissue oxygenation, reduce inflammation, and enhance microcirculation, all of which could contribute to improved gingival health [14,15]. However, the studies also present limitations that hinder definitive conclusions. A fundamental limitation is the lack of standardization in ozone delivery methods and concentrations across studies [9-13]. All studies employed in-office delivery units or irrigators, limiting generalizability to home-based use, a significant advantage of chlorhexidine mouthwash, a common control intervention.

In addition, the volatile nature of ozone necessitates immediate preparation and poses challenges for storage, potentially reducing efficacy over time [10]. Developing practical storage solutions for ozonated water would be crucial for broader clinical applications. Another limitation is the absence of data on the optimal ozone concentration for effective treatment [9,10-13]. Determining this optimal concentration is essential for establishing evidence-based practice guidelines for ozone therapy in gingivitis management. The heterogeneity in delivery methods and ozone concentrations across studies limits the overall strength of the review's conclusions regarding the efficacy of ozonated water for plaque reduction. While some studies suggest promising antibacterial properties [10], others found chlorhexidine more effective in reducing plaque index [11]. This inconsistency necessitates further investigation with standardized protocols. The limitations identified within the included studies also translate to limitations within this review. The lack of data on optimal ozone concentration and practical storage solutions restricts our ability to definitively assess the potential of ozonated water as a mainstream treatment option for gingivitis. Future research should prioritize large-scale, long-term clinical trials employing standardized ozone concentrations and delivery methods. These trials should determine the optimal ozone concentration for effectively treating gingivitis and associated pathogens. In addition, research efforts should focus on developing practical and user-friendly storage solutions for ozonated water to facilitate broader clinical applications.

## Conclusions

Although this systematic review reveals promising initial evidence for ozonated water in reducing gingival bleeding, it highlights the need for further exploration. Well-designed, large-scale trials with standardized ozone concentrations and delivery methods are crucial to definitively assess efficacy and determine the optimal concentration against gingivitis-related pathogens. Developing practical storage solutions for ozonated water would further facilitate its potential as a safe and natural alternative for managing gingivitis.
